# Long-term investigation of environmental radioactivity levels and public health around the Qinshan Nuclear Power Plant, China

**DOI:** 10.1038/s41598-022-09091-2

**Published:** 2022-03-23

**Authors:** Yiyao Cao, Junping Lin, Kangle Zhai, Wei Jiang, Hua Zou, Hong Ren, Peng Wang, Xiangjing Gao, Meibian Zhang, Shunfei Yu, Yaoxian Zhao, Zhiqiang Xuan, Dongxia Zhang, Yulian Liu, Xiaoming Lou

**Affiliations:** 1 Department of Occupational Health and Radiation Protection, Zhejiang Provincial Center for Disease Control and Prevention, Hangzhou, 310051 Zhejiang China; 2 Institute of Radiation Medicine, Chinese Academy of Medical Science and Peking Union Medical College, Tianjin, 300192 China; 3 National Institute for Communicable Disease Control and Prevention, Chinese Center for Disease Control and Prevention, Beijing, 102206 China; 4 Center for Disease Control and Prevention of Haiyan, Jiaxing, 314300 Zhejiang China; 5 National Institute of Occupational Health and Poison Control, Chinese Center for Disease Control and Prevention, Beijing, 100050 China

**Keywords:** Environmental chemistry, Environmental monitoring

## Abstract

To evaluate the impact of the Qinshan Nuclear Power Plant (Qinshan NPP) in normal operation on the surrounding environment and population, the radioactivity levels of drinking water and the ambient environment, as well as the residents’ cancer incidence, were continuously monitored for a period of 9 years (2012–2020). All of the gross *α* and *β* radioactivity concentrations in drinking water were less than the WHO recommended values (0.5 Bq/L for gross *α* and 1 Bq/L for gross *β*). The results of ambient environment accumulated dose monitored by thermoluminescent dosimeters (TLDs) indicated that the ambient environment radioactive level around the Qinshan NPP is consistently at natural background radiation levels. The age-dependent annual effective doses due to the ingestion of tap water or exposure to the outdoor ambient environment are lower than the reference dose of 0.1 mSv/year. The corresponding excess risks are at relatively low levels. Thus, the consumption of drinking water and outdoor activities are not expected to give rise to any detectable adverse effects on the health of the public around the Qinshan NPP. For all cancers combined, the age-standardized incidence rate by the Chinese 2000 standard population of the inhabitants living around Qinshan NPP is consistent with that of Zhejiang Province as a whole. Based on current radiation risk estimates, radiation exposure is not a plausible explanation for any excess cancers observed in the vicinity of the Qinshan NPP.

## Introduction

Nuclear power is a type of clean, efficient, and low-carbon energy that plays an important role in meeting future energy needs and addressing global climate change^1^. However, from the perspective of the public, nuclear power is still controversial energy and has many vulnerable characteristics in the aspect of nuclear safety because nuclear power plants (NPPs) are a potential source of radioactive pollution in the environment^2^. Radiation exposure is also considered a carcinogenic factor and there is ample evidence of increased cancer risk in humans at doses above 100 mSv^3^. Although there is no evidence of increased cancer risk for doses less than 100 mSv, it is assumed that the relationship between dose and health risk is linear with even trivial doses carrying small increases in risk^4^.

With the rapid expansion of nuclear power in recent years, China currently has 51 nuclear power reactors in operation and 13 reactors under construction, which are mainly located in the eastern and southern regions^5^. Approximately 100 million people live within 30 km of NPPs^6^. Thus, public concerns have arisen about the impacts of NPPs on the local environment and health. Accidents that have taken place at NPPs have increased the public’s concerns over radioactive pollution and malignant tumours induced by radiation exposure and decreased public acceptance of nuclear power, especially after the Fukushima nuclear accident^2,7^.

China has maintained a good nuclear safety record for a long time. By June 2019, the industry had operated safely and stably for more than 300 reactor-years, and there had been no incidents or accidents at or above Level 2 under the International Nuclear and Radiological Event Scale (INES)^8^, which provides a numerical rating (seven levels in all) that indicates the significance of nuclear or radiological events^9^. The incidence of Level 0 deviations and Level 1 anomalies had also decreased^8^. In the comprehensive ranking of similar units of the World Association of Nuclear Operators (WANO) in recent years, operating units in China have performed above the world median for more than 80 percent of the indicators, and have reached the world advanced level for more than 70 percent of the indicators^8^.

The impacts of NPPs during operation have been studied in China^10–15^ and many other countries^16–19^. Some of these studies focus on radioactive levels of environmental samples, such as drinking water, food, soil and air, and radiation doses of people living around NPPs, while other studies focus on population health risk and cancer incidence. However, comprehensive studies are rarely available in the literature.

The Qinshan NPP was the first NPP designed and constructed indigenously by China and has been in use since 1991. The NPP is located in Haiyan, a county of Zhejiang Province. There are two types of radiation exposure for members of the public around NPPs: internal and external radiation exposure. The ingestion of drinking water is one of dose contributor for internal radiation exposure. And exposure to the ambient environment represents the dominant pathway for external radiation exposure^20^. The annual effective dose (*AED*), which is a radiation protection quantity, has been considered a useful tool for radiation exposure risk assessment and policy-making on radioactive pollution^21^. To evaluate the radiological impact of the Qinshan NPP on the environment and people and possible radioactive pollution, the radioactivity levels (i.e., total alpha and beta) of drinking water samples and the ambient environment were continuously monitored for a period of 9 years (2012–2020). Subsequently, the long-term trends of environmental radioactivity were analysed; the age-dependent *AED* and health risk derived from the ingestion of drinking water as well as external exposure from the ambient environment were estimated. In addition, the cancer incidence of the residents was investigated.

The main objective of this paper is to present baseline data on the environmental radioactive levels and cancer incidence around the Qinshan NPP. The data may be helpful to provide a scientific basis for decision-making on radioactive monitoring management and public acceptance about NPPs. Moreover, a pre-accident health baseline is required to evaluate the public health consequences once an accident occurs at the NPP. Compared to previous studies, the present study has the following characteristics: (1) The long-term trends of environmental radioactivity levels around the Qinshan NPP are firstly assessed based on environmental monitoring data from 2012 to 2020. (2) To the best of our knowledge, this is the first time that the annual effective dose (*AED*) has been specially calculated with Chinese environmental exposure factors that consider age and regional variability. (3) The incidence and the temporal trends of cancer incidence of radiation-sensitive cancers was analyzed specially to reveal the impact of the Qinshan NPP in normal operation on the health of people living around it. (4) This is a comprehensive study involving long-term monitoring of radioactive levels, radiation dose calculation, health risk estimation, and cancer incidence analysis in the vicinity of NPPs in China, which will provide a more comprehensive understanding of the impact of nuclear power plants on the surrounding environment and population.

## Material and methods

### Monitoring of radioactivity levels of drinking water samples

In this study, the radioactivity concentrations of gross *α* and gross *β* were monitored to determine the radioactivity levels of drinking water samples. The World Health Organization (WHO) recommends the monitoring of gross *α* and *β* radioactivity concentrations in drinking water as the first step of the radiological aspect of determining drinking water quality because the process of identifying individual radionuclide radioactivity concentrations in drinking water is time-consuming and expensive, and the levels of gross *α* and *β* radioactivity can reflect the overall levels of radioactivity in drinking water^4^. The radioactivity concentration of gross *α* is an indicator of *α*-emitting radionuclides such as ^224^Ra and ^226^Ra, and gross *β* is an indicator of *β*-emitting radionuclides such as ^40^K and ^228^Ra^22^. Therefore, monitoring gross *α* and *β* radioactivity concentrations without regard to the identity of specific radionuclides is a practical approach that can be used to monitor the radioactive levels of drinking water samples^4^.

#### Sample collection and analysis of drinking water

In this study, drinking water samples were collected and analysed according to the standard examination methods for radiological parameters in drinking water by the National Health Commission of the People’s Republic of China and the Standardization Administration of China^23^.

Water samples were collected from three locations within 20 km around the Qinshan NPP. 

Table 1 shows a detailed description of the sampling sites which are representative of potential source locations of public exposure in this study. In the study region, source waters used in the local waterworks for supplying drinking water to the inhabitants are mainly from the reservoir called Qianmudang. The reservoir is connected to the Changshan river in the southwest of Haiyan and other nine ambient river water channels. The raw water from Qianmudang reservoir is treated by Sandi Waterworks. The treated water is called factory water. Then the factory water is transported through pipes to the residential areas. The water to drink from the residents’ faucet is called tap water. Each type of water sample was divided into two groups. One was collected in May (also called the dry season), and the other was collected in October (called the wet season). A 5-L volume of each drinking water sample was collected. The methods of collecting water samples are as follows:

1. Before sampling, use the water sample to be collected to wet the sampler, container and plug for 2–3 times.
2. Raw water: There were 2 sampling points: the bank and center of the reservoir. At each position, the sample was collected at 50 cm below the water surface in accordance with the crisscross method. And then mix them as one sample. During the time of sampling, it is important to avoid the disturbance of the water body and the mixing of substances floating on the water surface. When the situation such as the water rises or a turbidity flow occurs, the sampling is temporarily suspended.
3. Factory water: Before the treated water flows into the transport pipeline, collect 2–3 factory water in the storage tank. And then mix them as one sample.
4. Tap water: Collect tap water at the faucet in the residential area. At the time of sampling, in order to prevent mixing with the sediment which may be precipitated and deposited on the pipe at night, turn on the faucet and drain the water for a few minutes firstly, and then take a sample.
5. After sampling, add concentrated HNO_3_ to the water sample in the polyethylene bucket immediately according to the method of adding 20 ml of concentrated HNO_3_ per 1 L of water, so that the water sample is weakly acidic to avoid adsorption of radioactive substances on the wall of the container. And then cover the bucket tightly. Take them back to the laboratory for testing.
6. For the analysis, the upper clear water of the sample in the polyethylene drum is taken.

**Table 1:** Location information of sampling sites.

Sampling types	Sampling sites	Distance from the Qinshan NPP (km)	Locations information	
			Latitude	Longitude
Raw water	Qianmudang Reservoir	17.9	N 30° 33′ 14″	E 120° 49′ 53″
Factory water	Sandi Waterworks	18	N 30° 33′ 15″	E 120° 49′ 55″
Tap water	Haiyan country Centre for Disease Control and Prevention	8.9	N 30° 30′ 59″	E 120° 55′ 42″

In these ways, the experimental results could be avoided to be affected due to the collection or handling of water samples with sediments.

The radioactivity concentrations of gross *α* and *β* were measured using the *α*/*β* counting system. The models of *α*/*β* counters of the low-background multiple detectors were the BH1217II Four-channel Low-background *α*/*β* Measuring Instrument and the LB790 Ten-Channel Low-Background *α*/*β* Counter.

#### Quality assurance and quality control

Before determination of gross *α* and *β* radioactivity concentrations, the standard sources were used for efficiency calibration and correction, and the instruments were within the calibration cycle and qualified. The *α* standard source was a ^241^Am standard powder source, and the *β* standard source was a KCl (^40^K) standard powder source.

To control the measurement errors, 10% of the samples were analysed as parallel samples. The parallel sample measurements were within the error range tolerated. The lab participates in national gross *α* and *β* radioactivity intercomparison and proficiency testing organized by the Institute of Radiation Protection and Nuclear Safety Medicine of the Chinese Center for Disease Control and Prevention and acquires qualified results annually.

### Monitoring of radioactivity levels of the ambient environment

In this study, the ambient environmental accumulated dose, which is described by ambient dose equivalent H^*^(10), an operational quantity applied to area monitoring for assessing *AED* in people, was monitored to determine radioactivity levels of the ambient environment in the vicinity of the Qinshan NPP.

#### Sample collection and analysis

The ambient environmental accumulated dose was measured utilizing thermoluminescent dosimeters (TLDs). Two TLDs (LiF: Mg, Cu, P) were installed at a height of 2 m from the ground at every monitoring point in parallel. All TLDs were collected quarterly. Monitoring sites were set up uniformly within radii of 0–10 km, 10–20 km, and 20–30 km, with the nuclear power plant as the centre of the circle. Ten monitoring points are set in each scope, with a total of 30 monitoring sites throughout Haiyan County.

The analysis of ambient environmental accumulated dose was based on the Chinese national standard^24^: thermoluminescence dosimetry systems for personal and environmental monitoring, using a RGD-3B model thermoluminescent dosimeter reader. And the TLDs were calibrated with a Cs-137 source in accordance with the calibration procedure for detecting the ambient dose equivalent H^*^(10) in the Chinese standard JJG 593–2016: thermoluminescence dosimetry systems used in personal and environmental monitoring for X and γ radiation^25^.

#### Quality assurance and quality control

The TLDs were annealed with a thermoluminescent sophisticated annealing furnace before being installed to control the residual dose every time. The detection system was calibrated and qualified yearly by the Zhejiang Academy of Metrology. The lab participated in the nationwide ability assessment for personal external exposure dose monitoring organized by the Institute of Radiation Protection and Nuclear Safety Medicine of the Chinese Center for Disease Control and Prevention and acquires qualified results annually.

### Assessment of the long-term trends for environmental radioactivity levels

The trends of long-term environmental radioactivity levels were investigated to assess the variations of the drinking water and ambient environment around the operating NPP based on monitoring data: the gross *α* and *β* radioactivity and the ambient environmental accumulated dose. This statistical treatment method verifies whether changes exist quantitatively by comparisons of data from each season (for drinking water) or quarter (for the ambient environment). The Mann–Kendall verification method, a nonparametric test, was adopted in this study, which is regarded as suitable to verify whether the long-term environmental radioactivity levels are in natural fluctuation or if there are definite trends of change^26^. It determines the trend of the change via the computation of the Mann–Kendall test statistic ***Z***. At a significance level α of 0.05 for the test, if |***Z***|< ***Z***_1−α/2_, no monotonic trend exists. If ***Z*** > ***Z***_1−α/2_, an increasing trend exists. If ***Z*** < ***Z***_1−α/2_, a decreasing trend exists. The monitoring data were processed with Origin 2021 (Learning Version 9.8).

### Estimation of the annual effective dose and excess risk

For radiation protection, *AED* (mSv/year) is used to assess the risk to persons exposed to different forms of radiation: internal and external exposure. However, *AED* cannot be measured practically. Thus, the International Commission on Radiological Protection (*ICRP*) recommends the use of effective dose coefficients to convert the active concentration into *AED* for internal radiation resulting from the ingestion of radionuclides and ambient dose equivalent H^*^(10) to provide a conservative estimate of *AED* for external radiation^27^.

*AED* associated with internal exposure through ingestion of the drinking water was calculated by the following equation^21,22^:

1 $$AED_{i} = A \times C \times IR \times T$$

where *AED*_*i*_ is the annual effective dose caused by the ingestion of drinking water; *A* is the radioactivity concentration of gross *α* and *β* (Bq/L); *C* is the age-adjusted effective dose conversion factor for ingestion of radionuclides for members of the public (mSv/Bq); *IR* is the average daily ingestion rate of drinking water for groups with different ages and areas (L/d); and *T* is the duration of intake, which is 365.25 days.

Since gross *α* and *β* radioactivity are mainly given by ^226^Ra and ^40^K radioactivity, respectively^28^, the age-adjusted effective dose conversion factors of them provided by ICRP Publication 72^29^ were used to calculate the effective dose for gross *α* and *β*^30^.

The *IR* values of different age groups in Haiyan County, Zhejiang Province (Location of Qinshan NPP) obtained from research about environmental exposure related to activity patterns of the Chinese population was conducted by the Ministry of Ecology and Environment of the People’s Republic of China and are shown in Table 2^31,32^.

**Table 2:** The age-adjusted drinking water ingestion rates, outdoor occupancy factors and effective dose conversion factors.

Age (years)	1–2	2–3	3–4	4–5	5–6	6–9	9–12	12–15	15–18	≥ 18
*IR* (L/d)	0.101	0.899	0.773	0.755	0.822	0.904	0.952	0.981	1.094	1.588
O (d-1)	0.115	0.131	0.122	0.100	0.100	0.069	0.059	0.064	0.064	0.133
*C* of ^226^Ra (mSv/Bq)	9.6 × 10^–4^				6.2 × 10^–4^		8.0 × 10^–4^		1.5 × 10^–4^	2.8 × 10^–4^
*C* of ^40^K (mSv/Bq)	4.2 × 10^–5^				2.1 × 10^–5^		1.3 × 10^–5^		7.6 × 10^–6^	6.2 × 10^–6^

The ratios of the effective dose to the ambient dose equivalent *E*/H^*^(10) in *ICRP* 116 indicate that H^*^(10) is able to provide a reasonable assessment of *E* on the safe side^27^, which means that the ambient environmental accumulated dose monitored around the Qinshan NPP can be used to calculate the *AED* of the population resulting from exposure in the ambient environment^33^.

*AED* associated with external radiation through exposure in the ambient environment was calculated by the following equation:

2 $$AED_{e} = AD \times O$$

where *AED*_*e*_ is the annual effective dose caused by exposure in the ambient environment; *AD* is the ambient environmental accumulated dose (mSv); and *O* is the outdoor occupancy factor, which indicates the proportion of outdoor activity time of the population in the total activity time and is calculated from the outdoor activity time divided by the total activity time. The outdoor activity times for different age groups in Zhejiang, China, were collected from a research study focusing on environmental exposure related to activity patterns of the Chinese population^31,32^. The *O* values of different age groups in Haiyan are shown in Table 2.

The excess risk (*ER*), which refers to the excess rate of occurrence of a particular health effect associated with radiation exposure, was estimated using the following equation^33,34^:

3 $$ER = AED \times RF \times DL$$

where *AED* is annual effective dose; *RF* is detriment-adjusted nominal risk coefficients for cancer and heritable effects after exposure to radiation at a low dose rate (10^–5^/mSv) to express the severity of the consequence, which is 5.7 × 10^–5^/mSv (5.5 × 10^–5^/mSv for cancer and 0.2 × 10^–5^ /mSv for heritable effects); and *DL* is the duration of life, which is 70 years here.

### Analysis of cancer incidence

The demographic data and health data were obtained from the Zhejiang Provincial Chronic Disease Management System, which is coded using the International Classification of Diseases, Tenth Edition (ICD-10). Cancer incidence data were collected for all cancer sites combined, with a focus on leukaemia (ICD-10: C91-95) and cancers of the thyroid (ICD-10: C73). These two types of cancers are known to be particularly sensitive to radiation exposure^35^.

Then, a descriptive statistical analysis involving the overall incidence of malignant tumours, the sequence of cancer incidence, and the temporal trends of cancer incidence was conducted. The incidence of radiation-sensitive cancers was analysed. For comparisons of different age structures, the standardized cancer incidence was calculated adopting both the Chinese 2000 standard population and the WHO 2000 standard population as the basis. The temporal trends were characterized by annual percentage changes (APCs) and were estimated by the Joinpoint model. APC > 0 suggests an increasing trend, while APC < 0 suggests a decreasing trend. If 95% confidence intervals (95% CIs) did not include 0, the trend was considered statistically significant, and vice versa. All incidences and temporal trends were calculated by Joinpoint (Version 4.9.0.0; Statistical Methodology and Applications Branch, Surveillance Research Program, National Cancer Institute, Rockville, USA).

This study was carried out in accordance with the “Declaration of Helsinki” and approved by the Ethics Committee of Zhejiang Provincial Center for Disease Control and Prevention (CDC). The information provided by Chronic Disease Management System were kept confidential in Zhejiang CDC, and the ethics committee approved the permission to access the System and use the demographic data and health data because Zhejiang CDC has the authority of the Zhejiang provincial government to collect the cancer cases and related information, which is part of disease surveillance scope in Zhejiang CDC. And also, all methods were performed in accordance with the guidelines and regulations of Zhejiang CDC.

## Results and discussion

### Radioactivity concentrations of gross *α* and *β* of the drinking water sample and the long-term trends

The radioactivity concentrations of gross *α* and *β* for different types of drinking water samples around the Qinshan NPP from 2012 to 2020 are shown in Table 3.

**Table 3:** Radioactivity concentrations of gross *α* and *β* for different types of drinking water samples around the Qinshan NPP from 2012 to 2020 (× 10^–2^ Bq/L).

Year	Raw water		Factory water		Tap water	
	Gross *α*	Gross *β*	Gross *α*	Gross *β*	Gross *α*	Gross *β*
2012	2.6	19.1	0.8	22.1	1.6	28.6
2013	1.3	16.4	1.4	18.3	0.8	12.0
2014	4.1	20.5	1.3	15.9	1.7	15.2
2015	3.3	21.0	1.8	21.6	0.8	15.6
2016	0.8	16.5	0.8	13.7	1.5	14.5
2017	7.8	26.5	3.1	22.5	1.7	21.5
2018	0.8	23.0	0.8	18.5	0.8	7.2
2019	1.7	18.5	1.6	15.5	1.5	17.5
2020	1.4	11.1	0.8	15.4	1.3	22.5
Average	2.6 ± 2.2	19.2 ± 4.4	1.4 ± 0.8	18.2 ± 3.3	1.3 ± 0.4	17.2 ± 6.3

The gross *α* radioactivity concentrations determined from all types of drinking water samples from 2012 to 2020 range from 0.008 to 0.078 Bq/L, while the gross *β* radioactivity concentrations range from 0.072 to 0.286 Bq/L. The results of this study are generally consistent with previous studies: the findings of the pre-operational survey of the Sanmen NPP^36^ (more than 200 km from the Qinshan nuclear power plant) suggested that the gross *α* radioactivity concentrations of drinking water samples range from 0.001 to 0.063 Bq/L, while the gross *β* radioactivity concentrations range from 0.019 to 0.210 Bq/L. A study of gross *α* and *β* measurements in drinkable water from seven major geographical regions of China^21^ showed the mean values of the gross *α* radioactivity concentration (0.029 ± 0.041 Bq/L) and gross *β* radioactivity (0.091 ± 0.075 Bq/L) in the country as a whole, and the gross *α* radioactivity concentration (0.0204 ± 0.0321 Bq/L) and gross *β* radioactivity (0.0912 ± 0.0548 Bq/L) in East China (the location of the Qinshan NPP). The comparative results indicating that the radioactivity levels of drinking water in the vicinity of the Qinshan NPP are maintained at low, secure levels.

All of the radioactivity concentrations of gross *α* and *β* in this study are below the WHO recommended reference levels (0.5 Bq/L for gross *α*; 1.0 Bq/L for gross *β*), which means that the three types of water are acceptable for residents to consume from the perspective of radiological protection.

The gross *α* radioactivity concentrations for raw, factory, tap water samples have mean values of 0.026 ± 0.022 Bq/L, 0.014 ± 0.008 Bq/L, and 0.013 ± 0.004 Bq/L, respectively. The averages of the gross *β* radioactivity concentrations of the raw, factory, and tap water samples are 0.192 ± 0.044 Bq/L, 0.182 ± 0.033 Bq/L, and 0.172 ± 0.063 Bq/L, correspondingly. All of the radioactivity concentrations of gross *β* are larger than that of gross *α*. The rank order of radioactivity concentrations for both gross *α* and *β* is as follows: raw water > factory water > tap water. The gross *α* radioactivity concentrations of factory and tap water are significantly lower than those of raw water, which implies that the water treatment processes in waterworks are useful to reduce the radiation dose induced from the ingestion of water by decreasing the gross *α* radioactivity concentrations. These results are very meaningful. In general, radiation exposure due to gross *α* is of greater concern than that due to gross *β* because *α* particles impose a larger amount of radiation dose in the human body.

The difference of the gross *α* and *β* radioactivity concentrations of drinking water samples in different periods were analysed using Paired Samples *t*-test (SPSS 24.0). The results are shown in Table 4. In this study, there is no significant statistical difference between the dry and wet seasons. Some studies concluded that the gross *α* and *β* radioactivity concentrations are higher in the dry season than in the wet season for raw water samples due to the higher radioactive deposition during the dry season and the dilution effect of rainfall during the wet season^37,38^. Nevertheless, there is no significant climate change between the dry and wet season in Haiyan County, where the Qinshan NPP is located^39^, which perhaps accounts for the result.

**Table 4:** The difference of the gross *α* and *β* radioactivity concentrations of drinking water samples in different periods (× 10^–2^ Bq/L).

	Gross *α*		Gross *β*	
	Range	Mean ± SD^a^	Range	Mean ± SD
**Raw water**				
Dry season	0.8–7.4	3.0 ± 2.8	11.8–30.0	20.6 ± 5.8
Wet season	0.8–8.2	2.3 ± 2.5	4.2–31.0	17.8 ± 7.9
*p* value	0.505		0.452	
**Factory water**				
Dry season	0.8–5.4	1.5 ± 1.5	6.3–24.8	19.4 ± 6.3
Wet season	0.8–2.7	1.2 ± 0.8	6.5–25.2	16.9 ± 5.7
*p* value	0.573		0.463	
**Tap water**				
Dry season	0.8–2.6	1.2 ± 0.8	1.4–32.8	16.2 ± 9.9
Wet season	0.8–2.3	1.4 ± 0.7	8.1–24	18.2 ± 5.2
*p* value	0.665		0.348	

^a^*SD* standard deviation of the activity concentration.

The findings of the trend analysis by the monitoring data for three types of drinking water are shown in Table 5. All of the ***Z*** values are less than ***Z***_0.975_ = 1.976, which suggests that there is either an increasing or a decreasing trend during the period from 2012 to 2020.

**Table 5:** The trends of long-term radioactivity levels for drinking water.

	***Z*** value	Trend
**Raw water**		
Gross *α*	− 0.0086	No trend
Gross *β*	− 0.0129	No trend
**Factory water**		
Gross *α*	− 0.0057	No trend
Gross *β*	− 0.0445	No trend
**Tap water**		
Gross *α*	0.1510	No trend
Gross *β*	0.0700	No trend

### Ambient environmental accumulated dose and the long-term trends

The monitoring results of the ambient environmental accumulated dose around Qinshan NPP from 2012 to 2020 are shown in Table 6. The values of ambient environmental accumulated dose range from 0.244 to 0.603 mSv, with a mean value of 0.332 ± 0.111 mSv. The results of this study are in agreement with previously published findings, which were based on continuous supervision monitoring of the environmental radioactivity level around the Qinshan NPP carried out by Zhejiang Province Radiation Environmental Monitoring since the operation of the Qinshan NPP was initiated from 1991 to 2011^40^. In their study, the average accumulated dose rate measured by TLDs in those two decades was 86.9 nGy/h, which was converted to an accumulated dose of 0.53 mSv on an annual basis; before the operation of the Qinshan NPP, the average accumulated dose rate was 109 nGy/h, corresponding to an accumulated dose of 0.67 mSv annually^40^. In addition, the results of the pre-operational investigation of the Sanmen NPP in the period from 2015 to 2017 showed that the ambient environmental accumulated dose range from 0.321 to 0.411 mSv^11^. The comparative results of the two studies demonstrate that the ambient environmental radioactive level around the Qinshan NPP in regular operation is consistently at natural background radiation levels and is not expected to increase during the three decades.

**Table 6:** Ambient environmental accumulated dose around the Qinshan NPP from 2012 to 2020.

Year	Ambient environmental accumulated dose^a^ (mSv)				
	1st quarter	2nd quarter	3rd quarter	4th quarter	The total
2012	0.066	0.082	0.059	0.037	0.244
2013	0.105	0.073	0.066	0.066	0.310
2014	0.087	0.076	0.075	0.052	0.290
2015	0.073	0.044	0.120	0.056	0.290
2016	0.078	0.058	0.076	0.066	0.278
2017	0.059	0.008	0.104	0.093	0.264
2018	0.211	0.122	0.167	0.103	0.603
2019	0.059	0.061	0.067	0.124	0.311
2020	0.059	0.182	0.104	0.057	0.402
Average	0.089 ± 0.048	0.078 ± 0.049	0.093 ± 0.035	0.073 ± 0.028	0.332 ± 0.111

^a^The data shown in the table is the average accumulated ambient radiation doses for all sampling points.

The calculation outcomes of the Mann–Kendall test for the quarterly monitoring data of 30 monitoring points during a period from 2012 to 2020 are ***Z*** = 1.30, ***Z***_0.975_ = 1.976, and |***Z***|< ***Z***_0.975_, which indicates that no monotonic trend exists.

The results of the trend analysis corroborate the inference that the ambient environment radioactive level in the vicinity of the Qinshan NPP fluctuates naturally and does not increase with the operation of the NPP.

### Age-dependent annual effective dose and excess risk

The main objective of the evaluation of the gross *α* and *β* radioactivity concentrations is to ensure that the *AED* caused by 1 year's consumption of drinking water will not exceed the reference dose level of 0.1 mSv/year, recommended by the WHO to guard against deleterious radiological health effects^4,41^. The results shown in Table 7 range from 3.9 × 10^–4^ to 9.3 × 10^–3^ mSv/year for the whole population from 2012 to 2020, suggesting that all of the calculated *AED* values are lower than the reference dose level.

**Table 7:** Age-dependent annual effective dose (*AED*_*i*_) and excess risk *ER*_*i*_ induced by the ingestion of drinking water (tap water) for the population around Qinshan NPP from 2012 to 2020.

Age (years)	*AED*_*i*_ (× 10^-3^ mSv/year)			*ER*_*i*_ × 10^–5^		
	Min	Max	Avg	Min	Max	Avg
1–2	0.39	1.05	0.73	0.16	0.42	0.29
2–3	3.51	9.30	6.47	1.40	3.71	2.58
3–4	3.02	8.00	5.56	1.21	3.19	2.22
4–5	2.95	7.81	5.43	1.18	3.12	2.17
5–6	1.94	4.97	3.50	0.78	1.98	1.40
6–9	2.14	5.46	3.85	0.85	2.18	1.54
9–12	2.55	6.02	4.39	1.02	2.40	1.75
12–15	2.63	6.21	4.53	1.05	2.48	1.81
15–18	0.70	1.89	1.30	0.28	0.75	0.52
> 18	1.56	3.79	2.73	0.62	1.51	1.09

The *AED* induced by the ingestion of water is related to the annual consumption volume of water, which varies by age and region^14^. In the previous studies, because of the shortage of data for the Chinese annual ingestion volume of drinking water, the WHO-recommended volume of drinking water for adults was employed for the calculation of *AED* regardless of the differences in age and area^10,14,42^. In this study, age-dependent annual effective dose (*AED*_*i*)_ was calculated and combined with detailed consumption volumes of different age groups in Haiyan. Comparing the average *AEDs* of different age groups, the 2–3-year-old had the largest value of 6.47 × 10^–3^ mSv, while the 1–2-year-old group had the smallest value of 0.73 × 10^–3^ mSv. Meanwhile, the corresponding *ERs* of *AEDs* for each age group are estimated in Table 8. The *ERs* for the whole population range from 1.6 × 10^–6^ to 3.71 × 10^–5^, which are below the recommended risk level of 3.99 × 10^–4^ derived from the reference dose level^4^.

**Table 8:** Age-dependent annual effective dose (*AED*_*e*_) and excess risk *ER*_*e*_ induced by the exposure of ambient environment for the population around Qinshan NPP from 2012 to 2020.

Age (years)	*AED*_*e*_ (× 10^–3^ mSv/year)			*ER*_*e*_ × 10^–5^		
	Min	Max	Avg	Min	Max	Avg
1–2	28.06	69.35	38.18	11.20	27.67	15.23
2–3	31.96	78.99	43.49	12.75	31.52	17.35
3–4	29.77	73.57	40.50	11.88	29.35	16.16
4–5	24.40	60.30	33.20	9.74	24.06	13.25
5–6	24.40	60.30	33.20	9.74	24.06	13.25
6–9	16.84	41.61	22.91	6.72	16.60	9.14
9–12	14.40	35.58	19.59	5.74	14.20	7.82
12–15	15.62	38.59	21.25	6.23	15.40	8.48
15–18	15.62	38.59	21.25	6.23	15.40	8.48
> 18	32.45	80.20	44.16	12.95	32.00	17.62

These results suggest that the health risk of the whole population caused by radiation exposure through the ingestion of drinking water is at a relatively low level, and from the perspective of radiation protection, tap water around the Qinshan NPP is quite safe to drink.

A statistical overview of *AEDs*, as well as *ERs* induced by exposure to the ambient environment for the population around the Qinshan NPP from 2012 to 2020, is presented in Table 8. The *AED* results range from 1.44 × 10^–2^ mSv/year to 8.02 × 10^–2^ mSv/year for the whole population from 2012 to 2020. The largest average *AED*, 4.416 × 10^–2^ mSv/year, is found in the > 18-year-old group, and the smallest, 1.959 × 10^–2^ mSv/year, is found in the 9–12-year-old group. The corresponding *ERs* are 1.762 × 10^–4^ and 7.82 × 10^–5^, respectively. According to the United Nations Scientific Committee on Radiological Effects estimates, the average *AED* per person received from terrestrial radiation (outdoors and indoors) ranges from 0.3 to 1 mSv, with an average of 0.48 mSv^35^. Thus, the *AED* caused by exposure to the ambient environment contributes to a tiny percentage of the total radiation dose and is within a reasonable scope.

The results of this study are lower than those of previous studies^10,43^ because the *AED* induced by exposure to the ambient environment is dependent on the proportion of outdoor activity time, namely, outdoor occupancy factors. The commonly used outdoor occupancy factor of 0.2 in previous studies may have overestimated the *AED* of the public around the Qinshan NPP.

### Cancer incidence in the vicinity of Qinshan NPP

#### Incidence of all cancer sites combined

From 2012 to 2020, a total of 14,075 new cases of malignant tumours were reported in the vicinity of Qinshan NPP, with a crude incidence rate of 412.12/100,000, an ASIRC (age-standardized incidence rate by Chinese 2020 standard population) of 221.35/100,000, and an ASIRW (age-standardized incidence rate by WHO 2000 standard population) of 211.17/100,000. Of these cases, 7,279 cases were males, with a crude incidence rate of 432.38/100,000, an ASIRC of 226.66/100,000, and an ASIRW of 216.33/100,000; 6796 cases were females, with a crude incidence rate of 392.43/100,000, an ASIRC of 225.20/100,000, and an ASIRW of 211.36/100,000. The ASIRC of inhabitants living around Qinshan NPP is consistent with that of the whole of Zhejiang Province^44^ (both sexes: 220.79/100,000; males: 220.05/100,000; females: 222.65/100,000). For all cancers combined, the ASIRC was stable over the study period (2012–2020) for males, while a slight upwards trend was observed for females (APC = 5.7%, 95% CI 3.7–7.8%). The detailed information is shown in Table 9.

**Table 9:** Cancer incidence around Qinshan NPP from 2012 to 2020(1/100,000).

Year	Males				Females				Both sexes			
	New cases	Crude rate	ASIRC^a^	ASIRW^b^	New cases	Crude rate	ASIRC	ASIRW	New cases	Crude rate	ASIRC	ASIRW
2012	722	388.90	225.49	219.42	568	299.80	178.79	169.44	1290	343.90	197.86	190.11
2013	718	386.27	216.19	212.37	562	295.53	173.10	164.16	1280	340.38	190.66	184.42
2014	820	439.94	238.26	236.06	747	391.07	227.69	215.24	1567	415.21	229.82	222.21
2015	805	430.85	233.27	224.62	681	355.11	207.98	195.20	1486	392.48	217.52	206.79
2016	808	431.70	224.36	220.40	767	398.56	219.32	208.20	1575	414.90	219.36	211.63
2017	814	433.63	211.22	205.09	794	410.73	234.10	218.43	1608	422.01	220.57	209.72
2018	822	437.35	220.61	211.77	820	422.70	239.61	224.10	1642	429.91	228.17	215.78
2019	918	488.47	235.27	226.06	908	466.73	273.78	252.71	1826	477.41	252.84	237.51
2020	852	453.38	208.87	202.33	949	486.52	276.12	258.18	1801	470.26	241.69	229.06
Total	7279	432.38	226.66	216.33	6796	392.43	225.20	211.36	14,075	412.12	221.35	211.17
APC (%)	–	2.2	− 0.5	− 0.7	–	6.2	5.7	5.4	–	4.1	2.8	2.5
95% CI (%)	–	0.9, 3.6	− 2.0, 1.0	− 2.2, 0.7	–	4.2, 8.3	3.7, 7.8	3.4, 7.5	–	2.6, 5.6	1.2, 4.4	0.9, 4.1

^a^Age-standardized incidence rate by Chinese 2000 standard population.

^b^Age-standardized incidence rate by WHO 2000 standard population.

**Figure 1:**
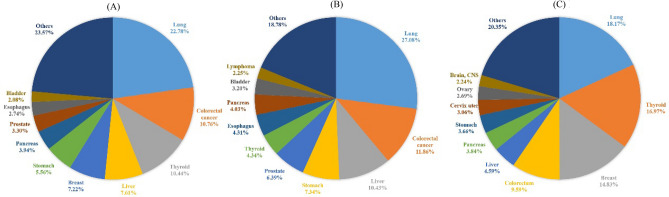
Distribution of cancer cases around Qinshan NPP from 2012 to 2020. (**A**) Both sexes, (**B**) males, and (**C**) females. For each sex, the area of the pie chart reflects the proportion of the total number of cases.

According to the crude incidence rate, the most common cancer for the residents living around Qinshan NPP is lung cancer, accounting for 22.78% of all new cancers in both sexes, 27.08% in males, and 18.17% in females. The 10 most commonly diagnosed cancers among men, in descending order, are cancers of the lung and colorectum, liver, stomach, prostate, thyroid, oesophagus, pancreas, bladder, and lymphoma, accounting for approximately four-fifths of all cancer cases. The corresponding cancers among women are lung, thyroid, breast, colorectum, liver, pancreas, stomach, cervix uterus, ovary, brain, and central nervous system cancers, accounting for nearly 80% of all cases (Table 10, Fig. 1).

**Table 10:** The rank of cancer incidence around Qinshan NPP from 2012 to 2020 (1/100,000).

Rank	Males				Females				Both sexes			
	Sites	Crude rate	ASIRC^a^	ASIRW^b^	Sites	Crude rate	ASIRC	ASIRW	Sites	Crude rate	ASIRC	ASIRW
1	Lung	117.08	56.32	55.32	Lung	71.31	36.81	44.35	Lung	93.87	45.55	44.35
2	Colorectum	51.26	25.41	25.16	Thyroid	66.58	51.16	44.84	Colorectum	44.36	21.30	20.95
3	Liver	45.09	23.00	22.24	Breast	58.21	36.92	33.92	Thyroid	43.01	33.68	29.17
4	Stomach	31.72	15.23	15.10	Colorectum	37.65	17.53	17.10	Liver	31.36	14.90	14.52
5	Prostate	27.62	12.65	12.36	Liver	18.02	7.59	7.64	Breast	29.75	18.74	17.22
6	Thyroid	18.77	16.01	13.36	Pancreas	15.07	6.13	6.20	Stomach	22.93	10.78	10.68
7	Esophagus	18.65	8.61	8.57	Stomach	14.38	6.76	6.64	Pancreas	16.22	7.16	7.13
8	Pancreas	17.40	8.26	8.09	Cervix uteri	12.01	8.04	7.15	Prostate	27.62	12.65	12.36
9	Bladder	13.84	6.76	6.57	Ovary	10.57	6.24	6.07	Esophagus	11.30	4.90	4.88
10	Lymphoma	9.74	5.43	5.26	Brain, CNS	8.78	5.56	5.35	Bladder	8.58	3.96	3.86

^a^Age-standardized incidence rate by Chinese 2000 standard population.

^b^Age-standardized incidence rate by WHO 2000 standard population.

#### Incidence of radiosensitive cancer

From 2012 to 2020, a total of 258 new leukaemia cases were reported in the vicinity of the Qinshan NPP, accounting for 1.83% of all cases, with a crude incidence rate of 7.55/100,000, an ASIRC of 5.22/100,000, and an ASIRW of 5.46/100,000. The ASIRC of leukaemia was stable over the period (APC = − 1.4%, 95% CI − 5.4 to 2.7%). From 2010 to 2014, the ASIRC of leukaemia in Zhejiang Province was 5.26/100,000, in line with the present study^45^ (Table 11). The results indicate that the normal operation of the Qinshan NPP has not yet caused an increase in the incidence of leukaemia for the population in the vicinity of the NPP.

**Table 11:** Leukaemia and thyroid cancer incidence around Qinshan NPP from 2012 to 2020 (1/100,000).

Year	Leukaemia				Thyroid cancer			
	New cases	Crude rate	ASIRC^a^	ASIRW^b^	New cases	Crude rate	ASIRC	ASIRW
2012	27	7.20	5.12	4.76	71	18.93	14.50	12.50
2013	30	7.89	6.81	7.86	100	26.59	20.78	18.14
2014	27	7.15	5.5	6.72	138	36.57	28.64	24.67
2015	24	6.34	5.29	4.95	115	30.37	23.16	20.11
2016	27	7.11	4.12	4.24	181	45.76	47.68	36.12
2017	29	7.61	4.76	5.37	167	43.83	36.65	30.68
2018	26	6.81	5.26	5.53	208	54.46	44.17	38.51
2019	31	8.1	5.18	5.27	230	60.13	48.79	42.07
2020	37	9.66	5.33	5.39	259	67.63	52.54	46.45
Total	258	7.55	5.22	5.46	1469	43.01	33.68	29.17
APC (%)	–	2.3	− 1.4	− 1.7	–	15.6	16.3	16.4
95%CI (%)	–	− 1.2, 5.8	− 5.4, 2.7	− 7.2, 4.2	–	11.3, 20.1	9.5, 23.5	10.7, 22.2

^a^Age-standardized incidence rate by Chinese 2000 standard population.

^b^Age-standardized incidence rate by WHO 2000 standard population.

A total of 1469 new thyroid cancer cases were reported, accounting for 10.44% of all new cancer cases, with a crude incidence rate of 43.01/100,000, an ASIRC of 33.68/100,000, and an ASIRW of 29.17/100,000. The ASIRC in females (16.01/100,000) was 3.2 times as high as that in males (51.16/100,000) (Table 10). The ASIRC of thyroid cancer increased dramatically by 3.62-fold between 2012 and 2020, from 14.50/100,000 to 52.54/100,000. Over the period, the temporal trend of the ASIRC of thyroid cancer in both sexes in the vicinity of the Qinshan NPP increased by 16.3% per year (95% CI 9.5–23.5%).

The ASIRC of thyroid cancer in this study is higher than that in a previous study in Zhejiang Province from 2010 to 2014 (24.11/100,000)^46^. This can be accounted for by the temporal trend of increasing thyroid cancer ASIRC both in Zhejiang Province^46^ (APC = 28.62%, 95% CI 21.00–36.72%) and nationwide in China^47^ (APC = 15.38%, 95% CI 13–16%). Many risk factors have been identified for thyroid carcinomas, such as ionizing radiation, iodine intake, female hormones, and body mass index (BMI)^48^. From this study, the radiation doses and the corresponding excess risks were too low to account for the increased number of thyroid cancers in the vicinity of the Qinshan NPP. The reason why the incidence of thyroid cancer has been growing is likely to be related to the availability and improvement of thyroid gland imaging examination techniques, such as thyroid ultrasonography, which has been incorporated into medical checkups for residents throughout Zhejiang Province, thus increasing the detection of thyroid cancer cases^46,48^. One of the possible other reasons is the rising rates of overweight and obesity in China^49^ because there is a linear dose–response relationship between BMI and thyroid cancer^50,51^.

Cancer incidence, especially radiosensitive cancers (leukaemia and thyroid cancer) of the population in the vicinity of NPPs, has been the topic of much scientific interest and public concern because NPPs are a potential source of radioactive material in the environment. Many studies have focused only on cancer incidence using epidemiological methods, which lack radiation exposure data on populations or simply use the distance of a residence from an NPP as a surrogate. However, it is important to know that radiation dose is essential to assess the effect of normally operational NPPs on cancer incidence among the residents of the surrounding area. In the present study, the long-term monitoring data of the gross *α* and *β* radioactivity concentrations of drinking water and the accumulated dose of ambient environment indicate that the radioactivity levels around the Qinshan NPP are maintained at natural background radiation levels. The resulting *AED* and *ER* are at fairly low and secure levels. Therefore, the operation of Qinshan NPP is not expected to contribute to an increase in the incidence of cancer among the surrounding population.

Although the incidence rates of thyroid cancer are high in the vicinity of the Qinshan NPP in this study, we argue that there are uncertainties in the conclusion that people living around the NPP have a higher risk of thyroid cancer. Further research may be necessary to clarify the association between thyroid cancer incidence and living near the NPP. Because the risk of radiation-induced thyroid cancer strongly depends on the exposure dose and age at exposure^18^, continuous monitoring of environmental radioactivity levels combined with well-designed cohort studies that are capable of controlling for potential confounding variables may provide a better understanding of the relationship.

In the future, more comprehensive environmental radioactivity monitoring, such as radioactivity in food and tritium radioactivity concentration in the environment generated from the heavy water reactor in the Qinshan NPP, is needed to determine radiation levels in the environment around the NPP and thus to assess the doses received by the population accurately. Continuous monitoring of the population is still required to evaluate the health state of the surrounding population considering the uncertainty of the long-term health effects of radiation exposure to low doses of radiation^52^.

## Conclusions

In this study, the radioactivity levels of drinking water samples and the ambient environment, as well as the residents’ cancer incidence in the vicinity of the Qinshan NPP, were investigated from 2012 to 2020. All of the gross *α* and *β* radioactivity concentrations were less than the WHO recommended values (0.5 Bq/L for gross *α* and 1 Bq/L for gross *β*), although variations were observed from different types of drinking water. The results of the ambient environment accumulated dose monitored by TLD dosimeters indicate that the environmental radioactive level around the Qinshan NPP is consistent with the natural background radiation levels. The analysis findings of the long-term trends assessment suggest that there are no trends in the monitoring items. The age-dependent *AEDs* due to the ingestion of tap water or exposure to the outdoor ambient environment are lower than the reference dose of 0.1 mSv/year. The corresponding *ERs* are at fairly low levels. Thus, the consumption of drinking water and outdoor activities are not expected to give rise to any detectable adverse effects on the health of the public around the Qinshan NPP. For all cancers combined, the age-standardized incidence rate by the Chinese 2000 standard population of the inhabitants living around Qinshan NPP is consistent with that of Zhejiang Province as a whole. No excess incidence of leukaemia was observed around the Qinshan NPP. The incidence of thyroid cancer is high, but it is also in line with the increasing trends in Zhejiang Province and all of China. Based on current radiation risk estimates, radiation exposure is not a plausible explanation for any excess cancers observed in the vicinity of the Qinshan NPP.
